# Multiple Lineages of Dengue Virus Serotype 2 Cosmopolitan Genotype Caused a Local Dengue Outbreak in Hangzhou, Zhejiang Province, China, in 2017

**DOI:** 10.1038/s41598-019-43560-5

**Published:** 2019-05-14

**Authors:** Hua Yu, Qingxin Kong, Jing Wang, Xiaofeng Qiu, Yuanyuan Wen, Xinfen Yu, Muwen Liu, Haoqiu Wang, Jingcao Pan, Zhou Sun

**Affiliations:** 1 Microbiology Laboratory, Hangzhou Center for Disease Control and Prevention, Hangzhou, Zhejiang Province 310021 China; 2 Institution of Infectious Disease Control, Hangzhou Center for Disease Control and Prevention, Hangzhou, Zhejiang Province 310021 China

**Keywords:** Infectious-disease diagnostics, Dengue virus, Viral infection

## Abstract

During July to November 2017, a large dengue outbreak involving 1,138 indigenous cases occurred in Hangzhou, Zhejiang Province, China. All patients were clinically diagnosed as mild dengue. Epidemiology investigation and phylogenetic analysis of circulating viruses revealed that at least three lineages of dengue virus serotype 2 (DENV-2) Cosmopolitan genotype initiated the outbreak during a short time. The analysis of the time to most recent common ancestor estimated that the putative ancestor of these DENV-2 lineages might rise no later than March, 2017, suggesting independent introductions of these lineages into Hangzhou. We presumed that group travelers visiting dengue-endemic areas gave rise to multiple introductions of these lineages during so short a time. Co-circulating of multiple DENV-2 lineages, emerging of disease in urban areas, hot and humid weather in Hangzhou adequate for mosquito breeding, and limited dengue diagnosis abilities of local hospitals, were the reasons causing the large local outbreak in Hangzhou.

## Introduction

Dengue is a mosquito-borne infectious disease caused by dengue viruses (DENVs) which consists of four antigenically distinct but closely related serotypes (DENV-1, DENV-2, DENV-3, and DENV-4). Dengue prevails in tropical and sub-tropical regions worldwide. It has been estimated recently that a total of 58.40 million symptomatic dengue viral infections, including 13.57 thousand fatal cases, occurred globally in 2013 (ref.^1^). In mainland China, the first confirmed dengue outbreak emerged in Guangdong Province in 1978. Since then, local dengue outbreaks have been frequently reported in some costal provinces of southeastern China (Guangdong, Guangxi, Hainan, Fujian and Zhejiang), and Yunnan Province of southern China, even in Henan Province in the centre part of China^2^. According to a nationwide surveillance data, a total of 69321 dengue infections and 11 deaths were reported in mainland China from 1990–2014 (ref.^3^). The highest annual case number was recorded up to 47056 in 2014, which was mainly contributed by a large local outbreak in Guangdong involving 45236 cases and 6 fatalities^4^. In general, local dengue outbreaks in China were considered to be caused by autochthonous transmission initiated by imported DENVs^3^; however, extensive phylogenetic analysis of DENVs circulating in Guangdong revealed that some lineages of DENV-1 strains persisted in consecutive years, suggesting the likelihood of dengue endemicity in the province^5,6^.

Zhejiang Province is not a dengue-endemic region, however imported dengue cases have been frequently reported during the past several decades^3^. Zhejiang features subtropical monsoon climate, presence of the vector of DENVs, *Aedes albopictus*, high density of human population, and more and more travelers visiting dengue-endemic regions, all of which provide basic conditions for the autochthonous transmission of imported DENVs. Since 2004, local dengue outbreaks have been identified in several cites of Zhejiang Province, including Ningbo City (DNEV-1)^7^, Yiwu City (DNEV-3)^8^, Wenzhou City (DNEV-1)^9^, and Shaoxin City (DNEV-2)^10^. During July to November 2017, the largest dengue outbreak in Zhejiang occurred in Hangzhou City, the capital of Zhejiang Province, and >1000 indigenous dengue cases were recorded. Recently Yan *et al*. also reported that 1149 indigenous and 80 imported cases occurred in Zhejiang in 2017, with almost all of indigenous cases being found in Hangzhou City^11^. Here, to trace the origin of the pathogen and reveal the reasons causing such a large outbreak in Hangzhou, we conducted a comprehensive investigation combining an epidemiology survey enhanced with geographical epidemiology information and a robust phylogenetic analysis with a number of sequences of circulating viruses.

## Results

### Local outbreak identification

Since dengue surveillance was started in the middle of 2000s, local outbreak of dengue had not been found in Hangzhou until this outbreak occurred. Although imported dengue cases have been frequently reported recently in Hangzhou, no indigenous case has been identified so far, except one found at Xiaoshan District, Hangzhou, in 2014. On August 22, 2017, an indigenous dengue case (the index patient of the outbreak) was reported by Shangcheng District Center for Disease Prevention and Control in Hangzhou. The patient got a fever, headache, and diarrhea on August 14, and his serum was positive for dengue IgM antibody. He managed a health club at Gongshu District and had no travel history to dengue-endemic region within 14 days before disease onset. An intensive epidemiology investigation was conducted immediately after the report was received. The sera of close contacts to the index patient (members and staff of the health club) were collected to test for dengue IgM antibody. On August 22, a total of 14 contacts were found to be dengue IgM positive. In these IgM positive contacts, only one was asymptomatic. The earliest onset date among them was July 15, suggesting that dengue had circulated in the members and staff of the health club for quite a while before the index patient was found. The patient with the earliest onset date had also no travel history to any dengue-endemic region. However, it was found that 11.96% (145/1212) of the club members had overseas travel histories, and 44.83% (65/145) of their destinations were Southeast Asia countries where dengue is endemic.

**Figure 1:**
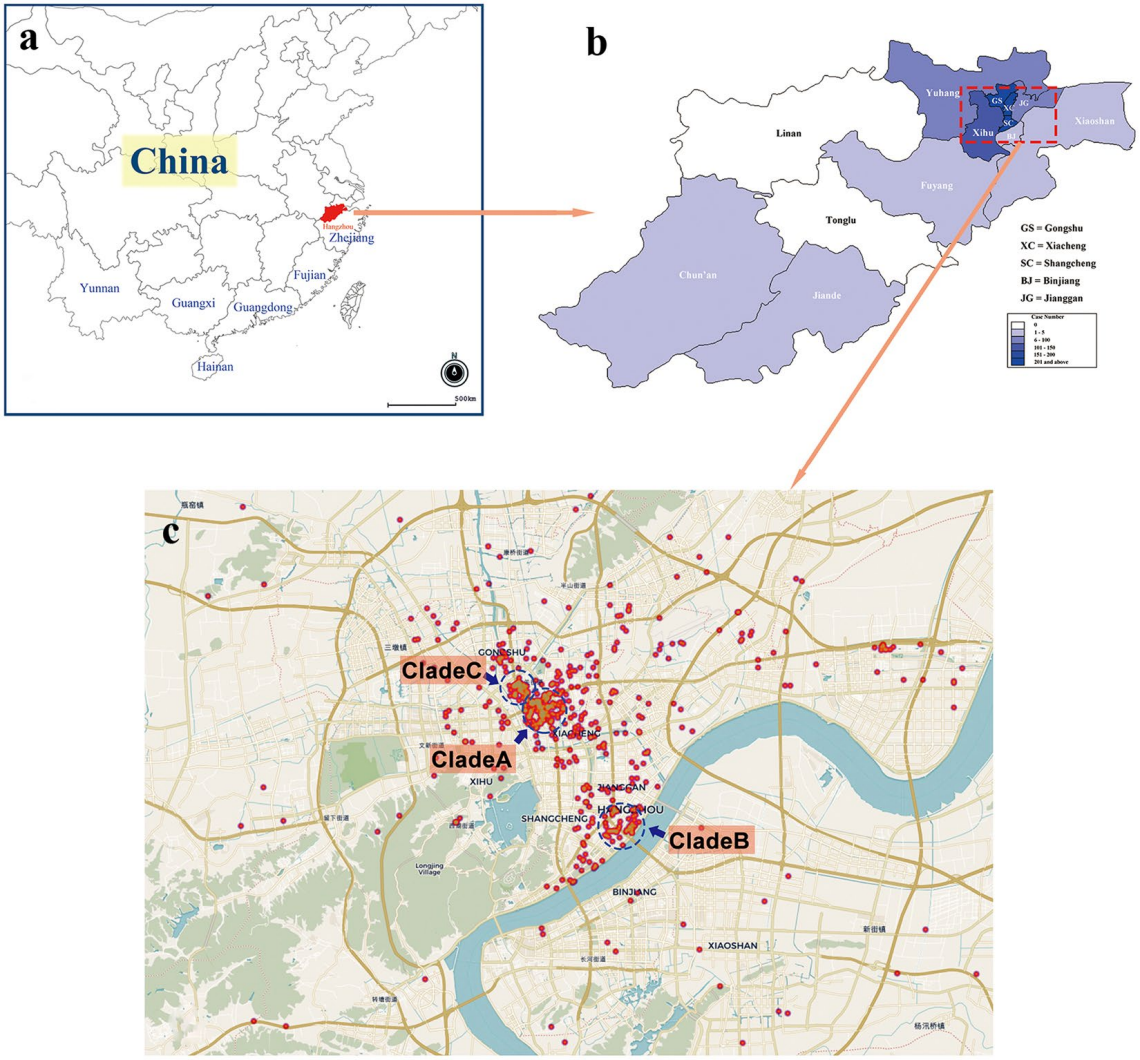
The geographical epidemiology information of the local dengue outbreak in Hangzhou. The locations of Hangzhou City, Zhejiang Province, Fujian Province, Guangdong Province, Guangxi Zhuang Autonomous Region, Yunan Province, and Hainan Province were showed in (**a**). The districts or counties of Hanghzou were marked in the sketched map in (**b**) and the depths of color represent the dengue case numbers occurring in the districts or counties. The panel C is a scatter plot of the dengue cases in the regions where the outbreak started and most cases were found, including Gongshu District, Xiacheng District and Shangcheng District. Three probable starting spots of the outbreak were pinpointed using blue circles. The maps in (**a**,**b**) were adapted from the EPI info (https://www.cdc.gov/epiinfo). Geographic distribution map of dengue cases (**c**) was created on the basis of the geodata of © OpenStreetMap contributors (https://www.openstreetmap.org).

**Figure 2:**
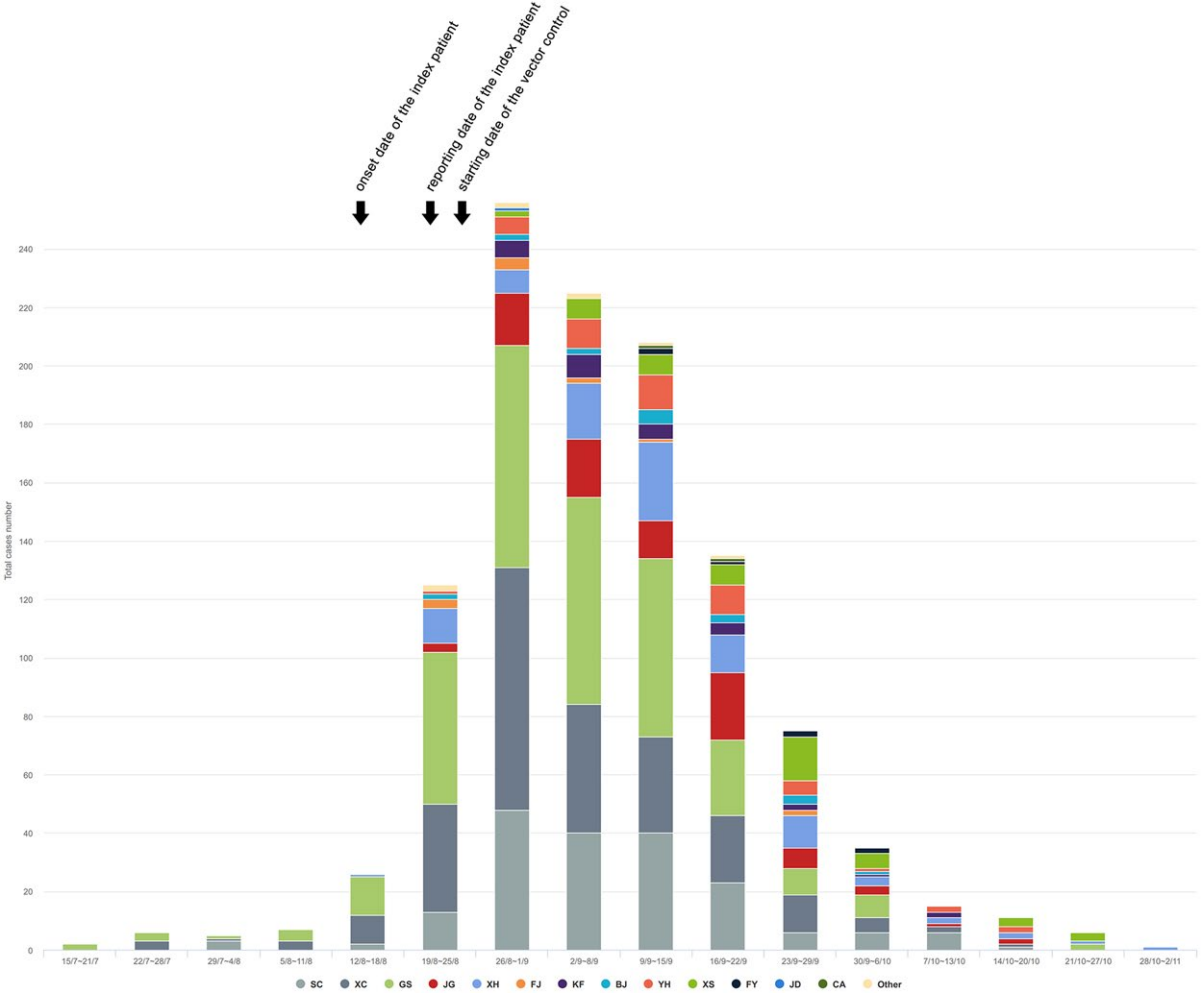
The numbers of indigenous dengue cases by week in Hangzhou from July 15 to November 2. The colors represent the districts or counties of Hangzhou where dengue patients occurred. SC: Shangcheng District; XC: Xiacheng District; GS: Gongshu District; JG: Jianggan District; XH: Xihu District; FJ: Xihu Fengjing District; KF: Xiasha District; BJ: Binjiang District; YH: Yuhang District; XS: Xiaoshan District; FY: Fuyang District; JD: Jiande City; CA: Chun’an County; Other: non-Hangzhou area.

Soon after the dengue outbreak linked to the health club was found, indigenous cases, clinically diagnosed or laboratory confirmed, emerged in succession in Gongshu District, Xiacheng District, Shangcheng District and Xihu District, as well as other seven districts or counties of Hangzhou (Figs 1 and 2). On August 24, an extensive community mobilization for mosquito control was organized by Hangzhou Municipal Government. Finally, the local outbreak lasted until November, and as of November 2, a total of 1138 indigenous cases, including 954 laboratory confirmed cases and 184 clinically diagnosed cases, were recorded. Among these indigenous patients, seven were visitors or travelers from non-Hangzhou dengue-free areas. All DENVs in 948 tested sera from indigenous cases were typed as DENV-2 by real-time RT-PCR. So, it can be concluded that the local outbreak was caused by DENV-2. The spatial and temporal distributions of the outbreak cases were showed in Figs 1 and 2.

During the period from July to November, 5 imported cases were identified also in Hangzhou. One DENV-2 infection (strain D2/CN/HZ-17/2017) which developed on June 30 originated in Malaysia. Two DENV-1 infections derived from Xishuangbanna, Yunnan Province, and 2 DENV-4 infections from Philippines.

### Demography and clinical features

The patient ages of the local dengue outbreak ranged between 1–95 with median at 51. The patients more than 50 years old accounted for 50.53% (575/1138). Male/female ratio is 1.004:1. Within the incubation period before disease onset, 0.61% (7/1138) had overseas travel history, and 8.44% (96/1138) had domestic travel history. 23.9% (272/1138) patients claimed to be bitten by mosquito before disease onset. All patients were clinically diagnosed as mild dengue. Neither severe dengue case nor death was reported. The clinical features of 1138 cases with dengue infection were summarized in Table 1.

**Table 1:** Clinic features of 1138 indigenous cases with DENV-2 infection in the 2017 Hangzhou outbreak.

Features	No. of case	%
Fever	1107	97.3
Headache	699	61.4
Retro-orbital pain	209	18.4
Myalgia	574	50.4
Arthralgia	439	38.6
Rash	232	20.4
Small blood spots under skin	121	10.6
Asthenia	943	82.9
Vomiting	348	30.6
Blushing	282	24.8

### DENV RNA detection in mosquitoes

Approximate 2600 female adults of *Ae. albopictus* were captured from 8 districts in Hangzhou during the period from August to October. Among these mosquitoes, DENV RNA was detected in two samples (20 mosquitoes per sample) which were collected at Shangcheng District on August 28 and at Xihu District on August 26, respectively. Both DENV E gene sequence and genome sequence have been achieved in the sample from Shangcheng District (strain D2/Mosquito/CN/HZ-mos2/2017). Due to low virus loading in another sample, however, neither E gene sequence nor genome sequence has been determined.

### Phylogenetic analysis of DENV-2 E gene

**Figure 3:**
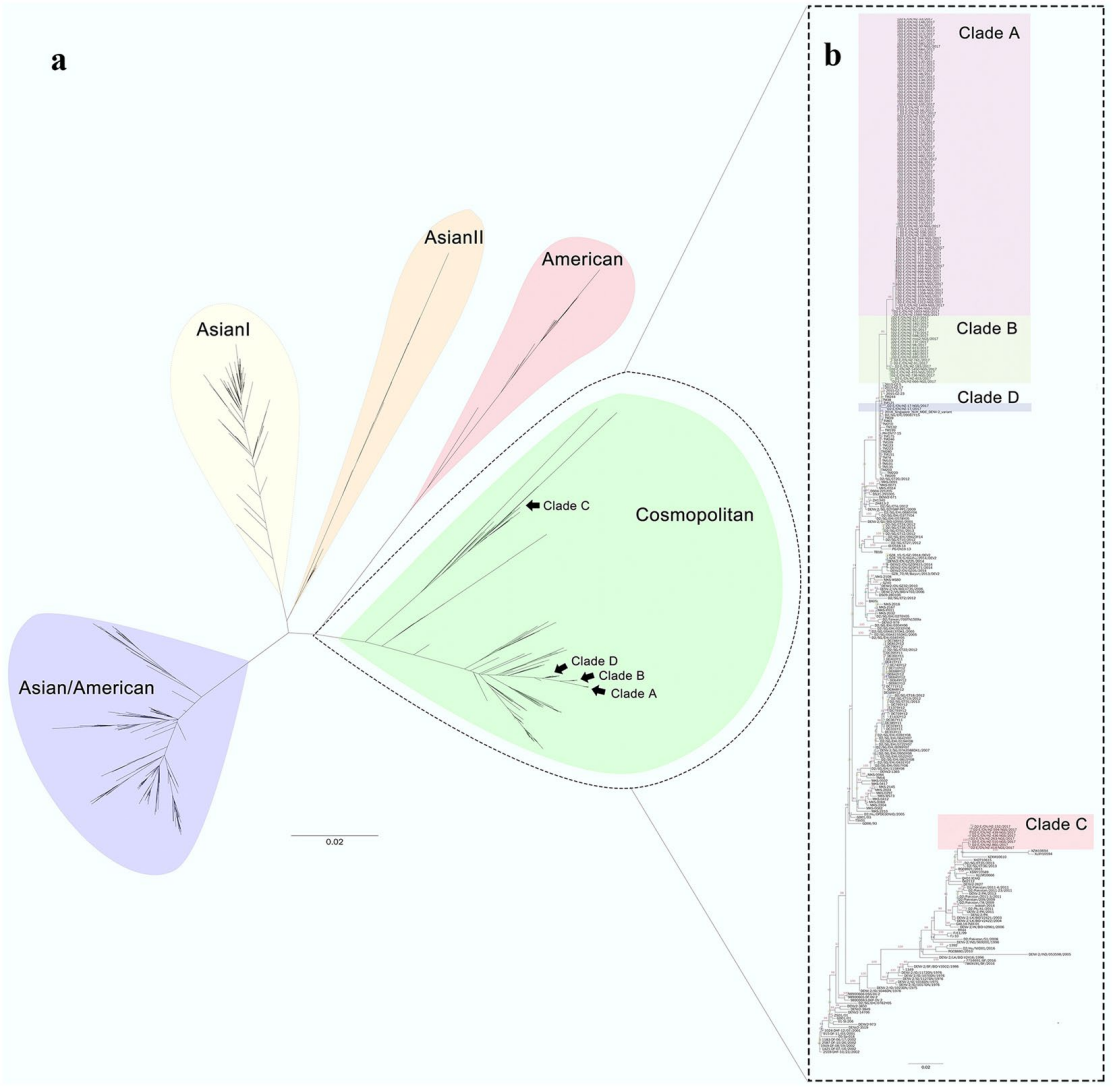
Phylogenetic analysis of DENV-2 E gene. The phylogenetic relationships of the Hangzhou strains and the representative strains of five DENV-2 serotypes across world were showed in (**a**). The phylogenetic tree in (**b**) presented the relationships of the Hangzhou strains and the DENV-2 Cosmopolitan genotype strains across world. The Hangzhou strains of clade A, clade B and clade C were from the indigenous patients, and the Hangzhou strain of clade D was from an imported case from Malaysia (strain D2/CN/HZ-17/2017). The strain from mosquito (strain D2/Mosquito/CN/HZ-mos2/2017) belonged to clade B. Bootstrap figures calculated by RAxML were showed at nodes of the tree.

All DENV-2 E gene sequences of 128 indigenous patients from the local outbreak, one imported case (strain D2/CN/HZ-17/2017), and one from the mosquito (strain D2/Mosquito/CN/HZ-mos2/2017) belonged to Cosmopolitan genotype and were clustered into 4 clades further, clade A, B, C and D (Fig. 3 and Supplementary Information file Table S1). Clade A consisted of 99 strains from the patients epidemiologically linked to the index patient at the health club of Gongshu District and the patients subsequently emerging at Gongshu District, Xiacheng District, Xihu District and others. Their onset dates ranged from August 16 to September 25. Clade B included 22 strains from the patients appearing mainly at Jinjiang street of Shangcheng District with the onset dates from August 20 to September 17. Clade C was made up of 8 strains from the patients arising at a residential district called Dongjia Xincun at Gongshu District with onset dates from August 25 to September 6. Clade D contained only the sequence from the imported case from Malaysia (strain D2/CN/HZ-17/2017). Thus, we found that at least three lineages of DENV-2 Cosmopolitan genotype caused this local outbreak in Hangzhou. Three start spots of the outbreak were located at Hangzhou urban areas, the health club at Gongshu District (clade A), Jinjiang Street at Shangcheng District (clade B), and Dongjia Xincun at Gongshu District (clade C) (Fig. 1).

**Figure 4:**
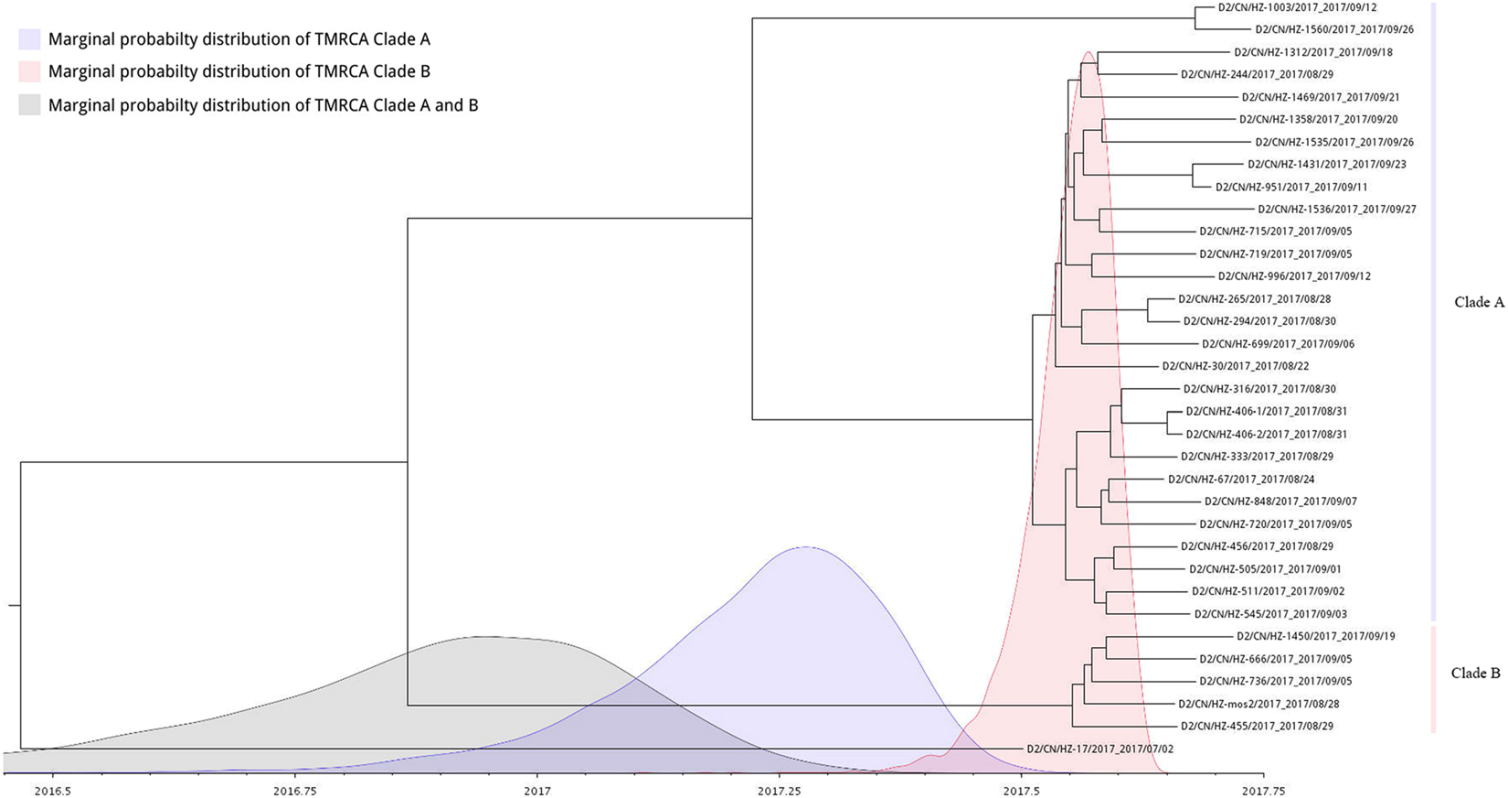
tMRCA analysis of the indigenous DENV-2 strains of clade A and clade B. Bayesian evolutionary trees of DENV-2 strains of clade A, clade B and clade D were constructed using BEAST2. Marginal probability distributions of tMRCA of different clades were calculated by Markov Chain Monte Carlo (MCMC) and showed in different colors.

On the phylogenetic tree, the clade A, B, and D were clustered closely. Especially both the clade A and B shaped their unique branches respectively. The close distance between the clade A and clade B suggested two possibilities: (1) the original strains of clade A and clade B were introduced independently from dengue endemic areas into Hangzhou and caused local outbreaks respectively via autochthonous transmission; (2) the ancestor of clade A and clade B was introduced into Hangzhou, then transmitted in patients with inapparent infection and evolved separately to form the outbreak strains of clade A and clade B. The identification of imported dengue case caused by the clade D strain of D2/CN/HZ-17/2017 seemed to support the latter possibility; however, there was probably insufficient time for the putative ancestor of clade A and clade B to form the initial strains of clade A and clade B via divergent evolution in Hangzhou. The time to most recent common ancestor (tMRCA) analysis estimated that mean tMRCA of clade A and clade B was 2016.866 (2016.3886~2017.2544, 95% highest posterior density interval) (Fig. 4). Thus the putative ancestor of clade A and clade B might rise no later than March, 2017, implying that it should emerge in dengue-endemic area rather than in Hangzhou. The tMRCA analysis also detected that under the strict clock, the mean nucleotide substitution rate of the outbreak strains was 3.08 × 10^−3^ substitutions per site per year (1.56 × 10^−3^~4.64 × 10^−3^, 95% highest posterior density interval) which was faster than those detected in other researches^12,13^. The driving factor of faster substitution in these DNEV-2 strains remains unknown.

The strains closest to the clade A and clade B included those circulating recently in Malaysia, Singapore, Philippines, Australia, and Guangdong, China, suggesting that Southeast Asia or Guangdong, China might be the origin of Hangzhou viruses of the clade A and clade B. The clade C was genetically distinct from the clade A, B and D, suggesting its independent introduction into Hangzhou. The strains close to the clade C came from Guangdong, Yunnan, Singapore, Vietnam, India, Pakistan, Sri Lanka, and Saudi Arabia.

### Phylogenetic analysis of DENV-2 genomes and their genomic characteristics

**Figure 5:**
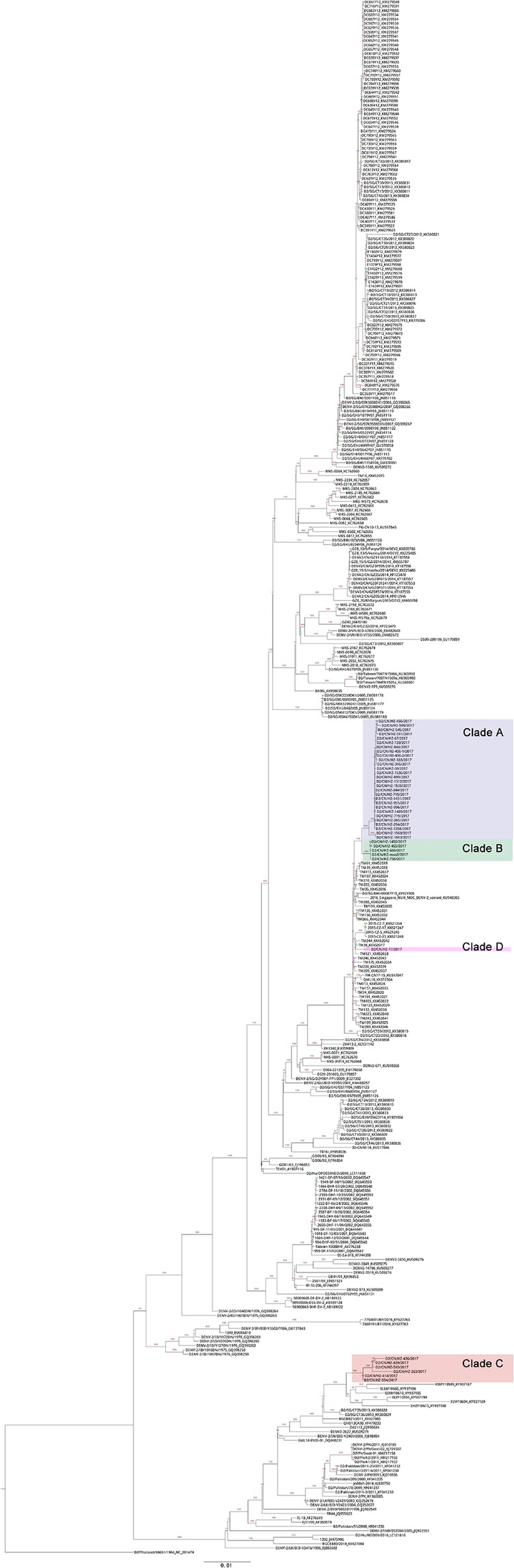
Phylogenetic analysis of DENV-2 Cosmopolitan genotype genomes. The Hangzhou strains were indicated with colors. The strains of clade A, clade B and clade C were from the indigenous patients of the Hangzhou outbreak, and the strain of clade D was from an imported case from Malaysia (strain Hangzhou/DF-17/2017). The strain from mosquito (strain Mosquito/Hangzhou/Mos-2/2017) belonged to clade B. Bootstrap figures calculated by RAxML were showed at nodes of the tree.

The phylogenetic structure based on the DENV-2 genomes was similar with that based on DENV-2 E genes. 40 DENV-2 genomes (39 from patients and one from mosquito) were also divided into 4 clades all of which belonged to Cosmopolitan genotype (Fig. 5 and Supplementary Information file Table S1). The DENV-2 genomes of clade A, B and D were clustered together, suggesting that they had a recent common ancestor. The genomes of clade C had a relatively remote distance to the genomes of clade A, B and D. The phylogentic relationships at the DENV-2 genome level also supported that the 2017 Hangzhou dengue outbreak was caused by multiple lineages of DENV-2 Cosmopolitan genotype, and there were at least three starting spots.

None of amino acid mutations known to be associated to increased DENV replication and viral fitness in hosts has been observed in the genomes of the 2017 Hangzhou DENV-2 strains (Supplementary Information file Table S2) as well as the DENV-2 genome of strain GZ05/2014 isolated in the Guangdong outbreak in 2014 (ref.^14^).

## Discussion

The 2017 dengue outbreak in Hangzhou is the largest historical outbreak in Zhejiang Province, involving a total of 1138 indigenous cases. To the best of our knowledge, the outbreak is the largest dengue outbreak in mainland China except Guangdong Province in the last decade. Yan *et al*. reported previously that in 2017 Zhejiang dengue outbreak, indigenous cases started at Gongshu District, Hangzhou, and then spread to the neighbor districts and outskirts, in some sera of which DENV-2 Cosmopolitan genotype was identified^11^. In this study, a number of viral sequences were employed in phylogenetic analysis, and our molecular epidemiological evidences both at the DENV E gene level and at the DENV genome level revealed that at least three lineages of DENV-2 Cosmopolitan genotype co-circulated during the outbreak. Moreover, combining the geographical epidemiological data and viral molecular data, we pinpointed three starting spots of the outbreak, the health club at Gongshu District, the Jinjiang street of Shangcheng District, and the Dongjia Xincun at Gongshu District, where strains of clade A, B, and C started to emerge respectively. However none of the original sources of the initial strains of clade A, B, or C has been identified through our epidemiological investigation. These starting spots were located at urban areas of Hangzhou featuring high density and frequent mobility of human population, which made disease spread easier and faster.

Although all three circulating DNEV lineages belongs to the same genotype, the genetic distances among these lineages suggest that the initial strains of these three lineages were introduced independently into Hangzhou. On the basis of the sequence similarities among the Hangzhou viruses and global viruses both at E gene level and genome level, it is deduced that the strains of clade A and clade B might originate from Southeast Asia or Guangdong, China. The strains very close to clade C were found in much wider geographical areas, including Guangdong and Yunnan of China, and countries in Southeast Asia, South Asia, and Central Asia, such as Singapore, Vietnam, India, Pakistan, Sri Lanka, and Saudi Arabia, suggesting that the lineage of DENV-2 circulated widely. According to the Statistical Yearbook of Hangzhou 2017 published by Hangzhou Municipal Government, the numbers of overseas tourists visiting Hangzhou and Hangzhou citizens traveling abroad reached to 3632252 and 1598969, respectively, in 2016. Frequent moving of tourists between Hangzhou and dengue-endemic areas provided the prerequisite to the occurrence of multiple introductions of dengue into Hangzhou during a short period. Due to the limited sequences of DENVs, especially those currently circulating in dengue-endemic areas, available in public nucleic acids databases, it is now impossible to determine the exact origins of the Hangzhou viruses through comparing viral sequence similarities.

It seemed to be too coincidental that three genetically close lineages belonging to the same genotype of DENV-2 started to circulate in a city during so short a period. Although molecular evidences suggest independent introductions of these DENV-2 lineages, there should be some relationship among these three origins of DENV-2s, especially between two very close lineages of clade A and clade B. All or two of initial sources of these 3 DENV-2 lineages might come from a limited range where dengue was endemic. Considering that group tourism is the most common way for Hangzhou citizens to travel abroad, we presume that the several group travelers might get infections independently during their stays at a dengue-endemic region, then they took the viruses back to their residences in Hangzhou and initiated multiple local circulations separately during a short time.

In the early phase of this outbreak, the numbers of dengue infections rose very quickly, and the disease spread to most counties and districts of Hangzhou soon, indicating that the viruses adapted to human and vector (*Ae. albopictus*) in Hangzhou quite well. However we did not find any amino acid mutation known to be associated with the change of viral virulence and replication in these viruses. The global distribution of the DENV-2 Cosmopolitan genotype implicates the high adaptability of the genotype in human and vectors. Moreover, Hangzhou’s hot and humid weather in summer was quite adequate for mosquito breeding, and the urban human population density of Hangzhou was high. Those facilitated autochthonous transmission of dengue. In addition, we found that there were many missing patients in contacts of the index patient of the outbreak, and their onset dates were earlier than one of the index patient, suggesting dengue had circulated for quite a while before the index patient was found. Limited dengue diagnosis abilities of local hospitals (if any patient had visited hospital) and mild symptoms should be the reasons causing the diagnosis missing of the initial dengue patients of the outbreak.

In Zhejiang history, no subsequent indigenous patient was found next year after dengue local outbreaks^7–10^, suggesting that dengue endemicity is not easy to establish in Zhejiang. Compared to these previous outbreaks, the Hangzhou 2017 outbreak involved more patients, larger region, and multiple lineages of viruses. Thus, it should be carefully monitored if these DENV-2 lineages could survive through winter in Hangzhou. Due to very frequent flow of tourists between Hangzhou and dengue endemic areas, future importation of dengue case should be inevitable in Hangzhou. In addition, there are a lot of cities similar with Hangzhou in China, so the Hangzhou outbreak might give us a warning that dengue is posing a serious threat to people living farther north than before in China. Comprehensive measures, including mosquito control to decrease vector densities, training to improve the dengue diagnosis abilities of local hospitals, and health education for tourists to prevent mosquito bike, should be taken to prevent future local outbreak in Hangzhou, or similar cities in China.

## Materials and Methods

### Epidemiology investigation and disease control

The epidemiology investigation was conducted according to the Guideline on Diagnosis and Therapy of Dengue Fever (Edition 2, 2014), issued by National Health and Family Planning Commission of the People’s Republic of China. The suspect cases were reported to local centers for disease prevention and control by doctors at hospitals in Hangzhou. The sera of suspect cases were collected to test for dengue virus nucleic acid and/or anti-dengue IgM antibody. The dengue patients included clinically diagnosed cases and laboratory confirmed cases which were identified on the basis of epidemiology history, clinic presents, and laboratory tests according to the guideline. The demographic, epidemiological and clinical data of dengue patients were collected. This study has been approved by the medical research review boards of Hangzhou Center for Disease Control and Prevention. Written informed consents were obtained from all participants and/or their legal guardians. Mosquito control was conducted according to the Guideline on Dengue Prevention and Control Techniques which was released by Chinese Center for Disease Prevention and Control.

### Mosquito trapping

Adult mosquitoes were captured using BG-Sentinel trap nearby the living places of dengue patients, then to be stored at −20 °C. Female adults of mosquitoes (*Ae. Albopictus*) were chosen to test for DENV RNA.

### Viral RNA extraction and real-time RT-PCR

For the patient’s sample, viral RNA was extracted from serum using QIAGEN RNeasy mini kit. For the mosquito sample, approximate 20 female mosquitoes (*Ae. albopictus*) were pooled in a centrifuge tube and homogenized at 30 per second for 1 min using Tissue Lyser II (Qiagen). Then 500 μl RTL was added into each tube for centrifugation at 12000 rpm for 10 min. The 200 μl supernatant each tube was used for viral RNA extraction in QIAcube (Qiagen) using QIAGEN RNeasy mini kit. The purified RNAs were used immediately or stored at −80 °C. One step real-time RT-PCR was applied to DENV RNA detection and typing using DENV detection and typing (serotype 1–4) kits (Shanghai ZJ Bio-Tech Co., Ltd., and bioPerfectus Technologies).

### IgM detection

Dengue IgM antibodies were tested in patient’s sera using Dengue IgM Kit (Panbio) according to the manufacturer’s protocol.

### DENV envelope (E) gene sequencing

DENV RNAs were extracted from acute phase sera of patients using QIAGEN RNeasy mini Kit, and cDNAs were synthesized using the SuperScriptTM III First-Strand Synthesis SuperMix (InvitrogenTM) according to manufacturer’s protocols. The cDNAs were stored at −80 °C until used. The E genes were amplified using Ex-Taq kit (TaKaRa) as described previously^15^. The primers used for E gene sequencing were listed in Supplementary Information file Table S3. The PCR products of DENV-2 E genes were sequenced on ABI PRISM 3700 Genetic Analyzer (Applied Biosystems) in Shanghai Sangon Co., Ltd.

### DENV genome sequencing

DENV-2 genome was amplified by PCR assays targeting 10 overlapping fragments covering the genome. The primers used for genome were presented in Supplementary Information file Table S3 (ref.^16^). All of 10 PCR products were mixed at the same molar concentration and purified by innuePREP PCRpure Kit. Prior to library preparation, the purified DNA was qualified and quantified using NanoDrop 2000 (Thermo-Fisher) and Qubit v3 (Thermo-Fisher). The DNA concentrations of all samples were diluted to 0.2 ng/μL as input DNA. Library was prepared by Nextera XT Library Kit (Illumina) and sequenced on an illumina Miseq (Illumina, San Diego, USA). FastQC (http://www.bioinformatics.babraham.ac.uk/projects/fastqc/) was applied to check the quality of raw reads, and Trimmamotic was used to trim low quality reads and adaptors^17^. All raw reads were assembled using SPAdes^18,19^ and mapped using BWA-SW^20^, SAMtools^21^, and VCFtools^22^.

### Phylogenetic analysis

The E gene and genome sequences of the reference DENV strains were obtained from the NIAID Virus Pathogen Database and Analysis Resource (ViPR) through the web site at https://www.viprbrc.org/. After removing the sequences with duplication, abnormal length or from sylvatic strains, multiple sequence alignments of DENV E genes and genomes were applied by MAFFT^23^, and the model of maximum likelihood tree was calculated by jModelTest^24,25^. The unrooted phylogenetic trees were constructed with RAxML (model GTR + I + G, bootstrap = 1000)^26^ and drew by figtree (http://tree.bio.ed.ac.uk/software/figtree/).

### tMRCA analysis

In order to conduct time-measured phylogenetic analysis, the datasets of genome sequences of clade A, B and D were used to generate input file within the BEAST2 (ref.^27^) software. The model selection was performed by model marginal estimates (MLE) log-likelihood through the path sampling (PS) and stepping-stone (SS) sampling methods. Rates of nucletides substitution and tMRCA were estimated using birth death serial skyline (BDSKY) method with three 100 M replicate runs of independent Markov Chain Monte Carlo (MCMC) chains which combined by LogCombiner with parameters and trees being sampled once every 10000 steps after removal of 10% burn in. MCMC outputs were diagnosed by tracer and the phylogeny tree was summarized by treeannotator. Figtree (http://tree.bio.ed.ac.uk/software/figtree/) was used to create the tMRCA phylogeny tree.

### Geographical epidemiology

The maps used to show the locations of districts and counties of Hangzhou City, Zhejiang Province and other coastal provinces where dengue has been frequently reported recently were adapted from the EPI info (https://www.cdc.gov/epiinfo) (Fig. 1a,b). Residential address of patients were translated into geo codes by GeoPy (https://github.com/geopy/geopy). CartoDB (https://github.com/CartoDB/cartodb) was used to create geographic distribution map of dengue cases based on the geodata of © OpenStreetMap contributors (https://www.openstreetmap.org) (Fig. 1c) under the Open Database License (https://www.openstreetmap.org/copyright).

## Supplementary information

Table S1, Table S2, Table S3, Table S5

Table S4

## Data Availability

The sequences of E genes and genomes of DENV-2 determined in this study have been deposited in the GenBank of the National Center for Biotechnology Information under the accession numbers listed in Supplementary Information file (Supplementary Information file Table S1) at Scientific Reports website. The reference sequences of DENV-2 E gene and genome downloaded from ViPR for phylogenetic analysis were listed in Supplementary Dataset (Table S4) and Supplementary Information file (Table S5) at Scientific Reports website.
