# Supplementary material for: Multiple Lineages of Dengue Virus Serotype 2 Cosmopolitan Genotype Caused a Local Dengue Outbreak in Hangzhou, Zhejiang Province, China, in 2017
Source: Sci Rep. 2019 May 14;9:7345. doi: 10.1038/s41598-019-43560-5 (PMC6517437; doi:10.1038/s41598-019-43560-5)
Supplement: Supplementary file 1 — Table S1, Table S2, Table S3, Table S5 [file 41598_2019_43560_MOESM1_ESM.pdf]

Supplementary Information

Multiple Lineages of Dengue Virus Serotype 2 Cosmopolitan Genotype Caused a Local Dengue Outbreak in Hangzhou, Zhejiang Province, China, in 2017

Hua Yu 1*， Qingxin Kong 2*， Jing Wang 2*， Xiaofeng Qiu 1， Yuanyuan Wen 2， Xinfen Yu 1， Muwen Liu 2， Haoqiu Wang 1, Jingcao Pan 1 §， Zhou Sun 2§

1. Microbiology Laboratory, Hangzhou Center for Disease Control and Prevention; 2. Institution of Infectious Disease Control, Hangzhou Center for Disease Control and Prevention, Hangzhou, Zhejiang Province, China, 310021.

* These authors contributed equally. § The correspondence should be addressed to Jingcao Pan (jingcaopan@sina.com) or Zhou Sun (hzcdc@qq.com).

Table S1. Accession Numbers of E gene sequences and genome sequences of dengue virus determined in this paper

Strain	Source	Type	Sequencing Method	Lineage (Clade)	Accession No.
D2/CN/HZ-1003/2017	Human blood	Genome	High-Throughput	A	MH110564
D2/CN/HZ-1312/2017	Human blood	Genome	High-Throughput	A	MH110565
D2/CN/HZ-1358/2017	Human blood	Genome	High-Throughput	A	MH110566
D2/CN/HZ-1431/2017	Human blood	Genome	High-Throughput	A	MH110567
D2/CN/HZ-1450/2017	Human blood	Genome	High-Throughput	B	MH110568
D2/CN/HZ-1469/2017	Human blood	Genome	High-Throughput	A	MH110569
D2/CN/HZ-1535/2017	Human blood	Genome	High-Throughput	A	MH110570
D2/CN/HZ-1536/2017	Human blood	Genome	High-Throughput	A	MH110571
D2/CN/HZ-1560/2017	Human blood	Genome	High-Throughput	A	MH110572
D2/CN/HZ-17/2017	Human blood	Genome	High-Throughput	D	MH110573
D2/CN/HZ-244/2017	Human blood	Genome	High-Throughput	A	MH110574
D2/CN/HZ-263/2017	Human blood	Genome	High-Throughput	C	MH110575
D2/CN/HZ-265/2017	Human blood	Genome	High-Throughput	A	MH110576
D2/CN/HZ-294/2017	Human blood	Genome	High-Throughput	A	MH110577
D2/CN/HZ-30/2017	Human blood	Genome	High-Throughput	A	MH110578
D2/CN/HZ-316/2017	Human blood	Genome	High-Throughput	A	MH110579
D2/CN/HZ-333/2017	Human blood	Genome	High-Throughput	A	MH110580
D2/CN/HZ-406-1/2017	Human blood	Genome	High-Throughput	A	MH110581
D2/CN/HZ-406-2/2017	Human blood	Genome	High-Throughput	A	MH110582
D2/CN/HZ-414/2017	Human blood	Genome	High-Throughput	C	MH110583
D2/CN/HZ-436/2017	Human blood	Genome	High-Throughput	C	MH110584
D2/CN/HZ-439/2017	Human blood	Genome	High-Throughput	C	MH110585
D2/CN/HZ-455/2017	Human blood	Genome	High-Throughput	B	MH110586
D2/CN/HZ-456/2017	Human blood	Genome	High-Throughput	A	MH110587
D2/CN/HZ-505/2017	Human blood	Genome	High-Throughput	A	MH110588
D2/CN/HZ-510/2017	Human blood	Genome	High-Throughput	C	MH110589
D2/CN/HZ-511/2017	Human blood	Genome	High-Throughput	A	MH110590
D2/CN/HZ-545/2017	Human blood	Genome	High-Throughput	A	MH110591
D2/CN/HZ-594/2017	Human blood	Genome	High-Throughput	C	MH110592
D2/CN/HZ-666/2017	Human blood	Genome	High-Throughput	B	MH110593
D2/CN/HZ-67/2017	Human blood	Genome	High-Throughput	A	MH110594
D2/CN/HZ-699/2017	Human blood	Genome	High-Throughput	A	MH110595
D2/CN/HZ-715/2017	Human blood	Genome	High-Throughput	A	MH110596
D2/CN/HZ-719/2017	Human blood	Genome	High-Throughput	A	MH110597
D2/CN/HZ-720/2017	Human blood	Genome	High-Throughput	A	MH110598

D2/CN/HZ-736/2017	Human blood	Genome	High-Throughput	B	MH110599
D2/CN/HZ-848/2017	Human blood	Genome	High-Throughput	A	MH110600
D2/CN/HZ-951/2017	Human blood	Genome	High-Throughput	A	MH110601
D2/CN/HZ-996/2017	Human blood	Genome	High-Throughput	A	MH110602
D2/Mosquito/CN/HZ-mos2/2017	Mosquito	Genome	High-Throughput	B	MH110603
D2/CN/HZ-1003/2017	Human blood	E gene	High-Throughput	A	MH110604
D2/CN/HZ-1312/2017	Human blood	E gene	High-Throughput	A	MH110605
D2/CN/HZ-1358/2017	Human blood	E gene	High-Throughput	A	MH110606
D2/CN/HZ-1431/2017	Human blood	E gene	High-Throughput	A	MH110607
D2/CN/HZ-1450/2017	Human blood	E gene	High-Throughput	B	MH110608
D2/CN/HZ-1469/2017	Human blood	E gene	High-Throughput	A	MH110609
D2/CN/HZ-1535/2017	Human blood	E gene	High-Throughput	A	MH110610
D2/CN/HZ-1536/2017	Human blood	E gene	High-Throughput	A	MH110611
D2/CN/HZ-1560/2017	Human blood	E gene	High-Throughput	A	MH110612
D2/CN/HZ-17/2017	Human blood	E gene	High-Throughput	D	MH110613
D2/CN/HZ-244/2017	Human blood	E gene	High-Throughput	A	MH110614
D2/CN/HZ-263/2017	Human blood	E gene	High-Throughput	C	MH110615
D2/CN/HZ-265/2017	Human blood	E gene	High-Throughput	A	MH110616
D2/CN/HZ-294/2017	Human blood	E gene	High-Throughput	A	MH110617
D2/CN/HZ-30/2017	Human blood	E gene	High-Throughput	A	MH110618
D2/CN/HZ-316/2017	Human blood	E gene	High-Throughput	A	MH110619
D2/CN/HZ-333/2017	Human blood	E gene	High-Throughput	A	MH110620
D2/CN/HZ-406-1/2017	Human blood	E gene	High-Throughput	A	MH110621
D2/CN/HZ-406-2/2017	Human blood	E gene	High-Throughput	A	MH110622
D2/CN/HZ-414/2017	Human blood	E gene	High-Throughput	C	MH110623
D2/CN/HZ-436/2017	Human blood	E gene	High-Throughput	C	MH110624
D2/CN/HZ-439/2017	Human blood	E gene	High-Throughput	C	MH110625
D2/CN/HZ-455/2017	Human blood	E gene	High-Throughput	B	MH110626
D2/CN/HZ-456/2017	Human blood	E gene	High-Throughput	A	MH110627
D2/CN/HZ-505/2017	Human blood	E gene	High-Throughput	A	MH110628
D2/CN/HZ-510/2017	Human blood	E gene	High-Throughput	C	MH110629
D2/CN/HZ-511/2017	Human blood	E gene	High-Throughput	A	MH110630
D2/CN/HZ-545/2017	Human blood	E gene	High-Throughput	A	MH110631
D2/CN/HZ-594/2017	Human blood	E gene	High-Throughput	C	MH110632
D2/CN/HZ-666/2017	Human blood	E gene	High-Throughput	B	MH110633
D2/CN/HZ-67/2017	Human blood	E gene	High-Throughput	A	MH110634
D2/CN/HZ-699/2017	Human blood	E gene	High-Throughput	A	MH110635
D2/CN/HZ-715/2017	Human blood	E gene	High-Throughput	A	MH110636
D2/CN/HZ-719/2017	Human blood	E gene	High-Throughput	A	MH110637
D2/CN/HZ-720/2017	Human blood	E gene	High-Throughput	A	MH110638
D2/CN/HZ-736/2017	Human blood	E gene	High-Throughput	B	MH110639

D2/CN/HZ-848/2017	Human blood	E gene	High-Throughput	A	MH110640
D2/CN/HZ-951/2017	Human blood	E gene	High-Throughput	A	MH110641
D2/CN/HZ-996/2017	Human blood	E gene	High-Throughput	A	MH110642
D2/Mosquito/CN/HZ-mos2/2017	Mosquito	E gene	High-Throughput	B	MH110643
D2/CN/HZ-100/2017	Human blood	E gene	Sanger	A	MH110644
D2/CN/HZ-102/2017	Human blood	E gene	Sanger	A	MH110645
D2/CN/HZ-103/2017	Human blood	E gene	Sanger	A	MH110646
D2/CN/HZ-104/2017	Human blood	E gene	Sanger	A	MH110647
D2/CN/HZ-105/2017	Human blood	E gene	Sanger	A	MH110648
D2/CN/HZ-106/2017	Human blood	E gene	Sanger	A	MH110649
D2/CN/HZ-107/2017	Human blood	E gene	Sanger	A	MH110650
D2/CN/HZ-108/2017	Human blood	E gene	Sanger	A	MH110651
D2/CN/HZ-109/2017	Human blood	E gene	Sanger	A	MH110652
D2/CN/HZ-110/2017	Human blood	E gene	Sanger	A	MH110653
D2/CN/HZ-111/2017	Human blood	E gene	Sanger	A	MH110654
D2/CN/HZ-113/2017	Human blood	E gene	Sanger	A	MH110655
D2/CN/HZ-115/2017	Human blood	E gene	Sanger	A	MH110656
D2/CN/HZ-1216/2017	Human blood	E gene	Sanger	A	MH110657
D2/CN/HZ-126/2017	Human blood	E gene	Sanger	A	MH110658
D2/CN/HZ-130/2017	Human blood	E gene	Sanger	A	MH110659
D2/CN/HZ-131/2017	Human blood	E gene	Sanger	A	MH110660
D2/CN/HZ-133/2017	Human blood	E gene	Sanger	A	MH110661
D2/CN/HZ-134/2017	Human blood	E gene	Sanger	A	MH110662
D2/CN/HZ-135/2017	Human blood	E gene	Sanger	A	MH110663
D2/CN/HZ-140/2017	Human blood	E gene	Sanger	A	MH110664
D2/CN/HZ-141/2017	Human blood	E gene	Sanger	A	MH110665
D2/CN/HZ-146/2017	Human blood	E gene	Sanger	A	MH110666
D2/CN/HZ-147/2017	Human blood	E gene	Sanger	A	MH110667
D2/CN/HZ-148/2017	Human blood	E gene	Sanger	A	MH110668
D2/CN/HZ-149/2017	Human blood	E gene	Sanger	A	MH110669
D2/CN/HZ-151/2017	Human blood	E gene	Sanger	A	MH110670
D2/CN/HZ-152/2017	Human blood	E gene	Sanger	C	MH110671
D2/CN/HZ-153/2017	Human blood	E gene	Sanger	A	MH110672
D2/CN/HZ-17/2017	Human blood	E gene	Sanger	D	MH110673
D2/CN/HZ-180/2017	Human blood	E gene	Sanger	B	MH110674
D2/CN/HZ-182/2017	Human blood	E gene	Sanger	B	MH110675
D2/CN/HZ-183/2017	Human blood	E gene	Sanger	B	MH110676
D2/CN/HZ-211/2017	Human blood	E gene	Sanger	A	MH110677
D2/CN/HZ-212/2017	Human blood	E gene	Sanger	B	MH110678
D2/CN/HZ-213/2017	Human blood	E gene	Sanger	A	MH110679
D2/CN/HZ-243/2017	Human blood	E gene	Sanger	A	MH110680

D2/CN/HZ-265/2017	Human blood	E gene	Sanger	A	MH110681
D2/CN/HZ-30/2017	Human blood	E gene	Sanger	A	MH110682
D2/CN/HZ-33/2017	Human blood	E gene	Sanger	A	MH110683
D2/CN/HZ-403/2017	Human blood	E gene	Sanger	B	MH110684
D2/CN/HZ-463/2017	Human blood	E gene	Sanger	B	MH110685
D2/CN/HZ-48/2017	Human blood	E gene	Sanger	A	MH110686
D2/CN/HZ-492/2017	Human blood	E gene	Sanger	A	MH110687
D2/CN/HZ-49/2017	Human blood	E gene	Sanger	A	MH110688
D2/CN/HZ-53/2017	Human blood	E gene	Sanger	A	MH110689
D2/CN/HZ-543/2017	Human blood	E gene	Sanger	A	MH110690
D2/CN/HZ-547/2017	Human blood	E gene	Sanger	B	MH110691
D2/CN/HZ-54/2017	Human blood	E gene	Sanger	A	MH110692
D2/CN/HZ-552/2017	Human blood	E gene	Sanger	A	MH110693
D2/CN/HZ-555/2017	Human blood	E gene	Sanger	A	MH110694
D2/CN/HZ-556/2017	Human blood	E gene	Sanger	A	MH110695
D2/CN/HZ-557/2017	Human blood	E gene	Sanger	A	MH110696
D2/CN/HZ-55/2017	Human blood	E gene	Sanger	A	MH110697
D2/CN/HZ-56/2017	Human blood	E gene	Sanger	A	MH110698
D2/CN/HZ-580/2017	Human blood	E gene	Sanger	A	MH110699
D2/CN/HZ-598/2017	Human blood	E gene	Sanger	B	MH110700
D2/CN/HZ-60/2017	Human blood	E gene	Sanger	A	MH110701
D2/CN/HZ-619/2017	Human blood	E gene	Sanger	B	MH110702
D2/CN/HZ-621/2017	Human blood	E gene	Sanger	B	MH110703
D2/CN/HZ-62/2017	Human blood	E gene	Sanger	A	MH110704
D2/CN/HZ-671/2017	Human blood	E gene	Sanger	A	MH110705
D2/CN/HZ-672/2017	Human blood	E gene	Sanger	A	MH110706
D2/CN/HZ-678/2017	Human blood	E gene	Sanger	A	MH110707
D2/CN/HZ-67/2017	Human blood	E gene	Sanger	A	MH110708
D2/CN/HZ-684/2017	Human blood	E gene	Sanger	A	MH110709
D2/CN/HZ-68/2017	Human blood	E gene	Sanger	A	MH110710
D2/CN/HZ-695/2017	Human blood	E gene	Sanger	B	MH110711
D2/CN/HZ-69/2017	Human blood	E gene	Sanger	A	MH110712
D2/CN/HZ-70/2017	Human blood	E gene	Sanger	A	MH110713
D2/CN/HZ-718/2017	Human blood	E gene	Sanger	A	MH110714
D2/CN/HZ-71/2017	Human blood	E gene	Sanger	A	MH110715
D2/CN/HZ-72/2017	Human blood	E gene	Sanger	A	MH110716
D2/CN/HZ-737/2017	Human blood	E gene	Sanger	B	MH110717
D2/CN/HZ-73/2017	Human blood	E gene	Sanger	A	MH110718
D2/CN/HZ-74/2017	Human blood	E gene	Sanger	A	MH110719
D2/CN/HZ-75/2017	Human blood	E gene	Sanger	A	MH110720
D2/CN/HZ-761/2017	Human blood	E gene	Sanger	B	MH110721

D2/CN/HZ-76/2017	Human blood	E gene	Sanger	A	MH110722
D2/CN/HZ-774/2017	Human blood	E gene	Sanger	B	MH110723
D2/CN/HZ-77/2017	Human blood	E gene	Sanger	A	MH110724
D2/CN/HZ-78/2017	Human blood	E gene	Sanger	A	MH110725
D2/CN/HZ-79/2017	Human blood	E gene	Sanger	A	MH110726
D2/CN/HZ-81/2017	Human blood	E gene	Sanger	A	MH110727
D2/CN/HZ-860/2017	Human blood	E gene	Sanger	C	MH110728
D2/CN/HZ-89/2017	Human blood	E gene	Sanger	A	MH110729
D2/CN/HZ-91/2017	Human blood	E gene	Sanger	B	MH110730
D2/CN/HZ-92/2017	Human blood	E gene	Sanger	B	MH110731
D2/CN/HZ-97/2017	Human blood	E gene	Sanger	A	MH110732
D2/CN/HZ-98/2017	Human blood	E gene	Sanger	B	MH110733
D2/CN/HZ-552/2017	Human blood	E gene	Sanger	A	MH110734

Table S2. Key amino acid mutations known to be associated with DENV virulence and viral replication in the genomes of the 2017 Hangzhou DENV-2 strains.

Strains	Clade	Protein position*																				
		E													NS1		NS4B		NS3	NS5		
		67	71	104	124	126	128	135	390	398	405	429	436	492	53	279	52	245	250	200	290	401
DF-17	D	N	A	G	N	E	K	L	S	I	T	F	L	M	G	F	L	N	E	K	T	R
DF-30	A	-	-	-	-	-	-	-	-	-	-	-	-	-	-	-	-	-	-	-	-	-
DF-67		-	-	-	-	-	-	-	-	-	-	-	-	-	-	-	-	-	-	-	-	-
DF-244		-	-	-	-	-	-	-	-	-	-	-	-	-	-	-	-	-	-	-	-	-
DF-265		-	-	-	-	-	-	-	-	-	-	-	-	-	-	-	-	-	-	-	-	-
DF-316		-	-	-	-	-	-	-	-	-	-	-	-	-	-	-	-	-	-	-	-	-
DF-333		-	-	-	-	-	-	-	-	-	-	-	-	-	-	-	-	-	-	-	-	-
DF-406-1		-	-	-	-	-	-	-	-	-	-	-	-	-	-	-	-	-	-	-	-	-
DF-406-2		-	-	-	-	-	-	-	-	-	-	-	-	-	-	-	-	-	-	-	-	-
DF-456		-	-	-	-	-	-	-	-	-	-	-	-	-	-	-	-	-	-	-	-	-
DF-505		-	-	-	-	-	-	-	-	-	-	-	-	-	-	-	-	-	-	-	-	-
DF-511		-	-	-	-	-	-	-	-	-	-	-	-	-	-	-	-	-	-	-	-	-
DF-545		-	-	-	-	-	-	-	-	-	-	-	-	-	-	-	-	-	-	-	-	-
DF-699		-	-	-	-	-	-	-	-	-	-	-	-	-	-	-	-	-	-	-	-	-
DF-715		-	-	-	-	-	-	-	-	-	-	-	-	-	-	-	-	-	-	-	-	-
DF-719		-	-	-	-	-	-	-	-	-	-	-	-	-	-	-	-	-	-	-	-	-
DF-720		-	-	-	-	-	-	-	-	-	-	-	-	-	-	-	-	-	-	-	-	-
DF-848		-	-	-	-	-	-	-	-	-	-	-	-	-	-	-	-	-	-	-	-	-
DF-951		-	-	-	-	-	-	-	-	-	-	-	-	-	-	-	-	-	-	-	-	-
DF-996		-	-	-	-	-	-	-	-	-	-	-	-	-	-	-	-	-	-	-	-	-

DF-1312		-	-	-	-	-	-	-	-	-	-	-	-	-	-	-	-	-	-	-	-	-
DF-1358		-	-	-	-	-	-	-	-	-	-	-	-	-	-	-	-	-	-	-	-	-
DF-1431		-	-	-	-	-	-	-	-	-	-	-	-	-	-	-	-	-	-	-	-	-
DF-1469		-	-	-	-	-	-	-	-	-	-	-	-	-	-	-	-	-	-	-	-	-
DF-1535		-	-	-	-	-	-	-	-	-	-	-	-	-	-	-	-	-	-	-	-	-
DF-1536		-	-	-	-	-	-	-	-	-	-	-	-	-	-	-	-	-	-	-	-	-
DF-1003		-	-	-	-	-	-	-	-	-	-	-	-	-	-	-	-	-	-	-	-	-
DF-1560		-	-	-	-	-	-	-	-	-	-	-	-	-	-	-	-	-	-	-	-	-
Mos-2		-	-	-	-	-	-	-	-	-	-	-	-	-	-	-	-	-	-	-	-	-
DF-455		-	-	-	-	-	-	-	-	-	-	-	-	-	-	-	-	-	-	-	-	-
DF-666	B	-	-	-	-	-	-	-	-	-	-	-	-	-	-	-	-	-	-	-	-	-
DF-736		-	-	-	-	-	-	-	-	-	-	-	-	-	-	-	-	-	-	-	-	-
DF-1450		-	-	-	-	-	-	-	-	-	-	-	-	-	-	-	-	-	-	-	-	-
DF-263		-	-	-	-	-	-	-	-	-	-	-	-	-	-	-	-	-	-	-	-	-
DF-414		-	-	-	-	-	-	-	-	-	-	-	-	-	-	-	-	-	-	-	-	-
DF-436	C	-	-	-	-	-	-	-	-	-	-	-	-	-	-	-	-	-	-	-	-	-
DF-439		-	-	-	-	-	-	-	-	-	-	-	-	-	-	-	-	-	-	-	-	-
DF-510		-	-	-	-	-	-	-	-	-	-	-	-	-	-	-	-	-	-	-	-	-
DF-594		-	-	-	-	-	-	-	-	-	-	-	-	-	-	-	-	-	-	-	-	-
		347	351	384	404	406	408	415	670	678	685	709	716	772	828	1054	2295	2488	1725	2691	2781	2892
		Polyprotein position																				

* The biological functions of these amino acid mutants referred to the following article: Zhao H, Zhao L, Jiang T, et al. Isolation and characterization of dengue virus serotype 2 from the large dengue outbreak in Guangdong, China in 2014. Sci China Life Sci. 2014. 57(12): 1149-55.

Table S3. Primers used in this research

Reaction	Primer name	Sequence(5'-3')	Reference
E Amplification	D2F	TTGAGACATCCAGGCTTCACC	1
E Amplification	D2R	CCACTATCGGCCTGCACCAT	
Reversering	D2-RE	AGAACCTGTTGATTCAACAGCACCAT	2
Genome Amplification	G1-F	AGTTGTTAGTCTACGTGGACCGACA	
Genome Amplification	G1-R	AGCACCATCTCATTGAAGTCGAGG	
Genome Amplification	G2-F	CAAACACTCCATGGTAGACAGAGG	
Genome Amplification	G2-R	TCCCRCTGCCACATTTCARTTCTTT	
Genome Amplification	G3-F	ACGCSAAGAAACAGGATGTYGTTGT	
Genome Amplification	G3-R	TCCTGCTGTTTGTGTATGGTAGCC	
Genome Amplification	G4-F	CACATGGAAGATGGAGAAAGCCTC	
Genome Amplification	G4-R	GCCGTRATTGGTATYGATACWGGAA	
Genome Amplification	G5-F	ATGGGMGTGACTTAYCTTGCCCTA	
Genome Amplification	G5-R	CTTCTCTAACTATGGCTGGAAGG	
Genome Amplification	G6-F	TGGTGTTGTCACAAGGAGTGGAG	
Genome Amplification	G6-R	TACGCCYTTCCRCCTGCYTCAG	
Genome Amplification	G7-F	GCAGACAGAARGTGGTGTTTTGATG	
Genome Amplification	G7-R	CATTAATACTTGAGTCACGCAGAGG	
Genome Amplification	G8-F	TTCAAGCAAAAGCAACCAGAGAAGC	
Genome Amplification	G8-R	CATGGTAWGCCCAYGTTTTGTATGG	
Genome Amplification	G9-F	CAGGAAGTGGATAGAACCYTRGCA	
Genome Amplification	G9-R	CATTACTGTGCCTCTTGGTGTTGG	
Genome Amplification	G10-F	CTGGTTGACAGGGAAAGAAATCTCC	
Genome Amplification	G10-R	AGAACCTGTTGATTCAACAGCACCAT	

Reference

1. Wang J, Chen H, Huang M, et al. Epidemiological and etiological investigation of dengue fever in the Fujian province of China during 2004-2014. Sci China Life Sci. 2017. 60(1): 72-80.
2. Cruz CD, Torre A, Troncos G, Lambrechts L, Leguia M. Targeted full-genome amplification and sequencing of dengue virus types 1-4 from South America. J Virol Methods. 2016. 235: 158-67.

Table S5. Information of DENV-2 genome reference sequences used in Figure 5

Strain	Accession	Genome Length	Year	Source	Country
01-St-206	KF744397	10176	2001	Homo sapiens	Philippines
05-Sa-018	KF744398	10176	2005	Homo sapiens	Philippines
1018-DF-12/03/2001	DQ645543	10671	12/03/2001	-N/A-	Taiwan
1024-DHF-12/07/2001	DQ645544	10671	12/07/2001	-N/A-	Taiwan
1183-DF-06/17/2002	DQ645545	10616	06/17/2002	-N/A-	Taiwan
1222-DF-06/24/2002	DQ645546	10671	06/24/2002	-N/A-	Taiwan
1349	EU056810	10723	1983	Homo sapiens	Burkina Faso
1392	JX475906	10670	2009	Homo sapiens	India
1421-DF-07/16/2002	DQ645547	10671	07/16/2002	-N/A-	Taiwan
1464-DHF-07/20/2002	DQ645548	10644	07/20/2002	-N/A-	Taiwan
1945-DHF-08/18/2002	DQ645549	10671	08/18/2002	-N/A-	Taiwan
1949-DF-08/19/2002	DQ645550	10671	08/19/2002	-N/A-	Taiwan
2015-CZ-17	KX621247	10681	2015	Homo sapiens	China
2015-CZ-23	KX621248	10681	2015	Homo sapiens	China
2015-CZ-5	KX621245	10681	2015	Homo sapiens	China
2015-CZ-7	KX621246	10681	2015	Homo sapiens	China
2016_Singapore_NUH_MDC_DENV-2_variant	KU948303	10707	02/23/2016	Homo sapiens	Singapore
2191-DF-09/12/2002	DQ645551	10671	09/12/2002	-N/A-	Taiwan
2208-DHF-09/13/2002	DQ645552	10671	09/13/2002	-N/A-	Taiwan
2559-DHF-10/23/2002	DQ645553	10671	10/23/2002	-N/A-	Taiwan
2587-DF-10/26/2002	DQ645554	10616	10/26/2002	-N/A-	Taiwan
2659-DHF-11/01/2002	DQ645555	10616	11/01/2002	-N/A-	Taiwan
2784-DF-11/18/2002	DQ645556	10671	11/18/2002	-N/A-	Taiwan
7754691/BF/2016	KY627763	10675	11/21/2016	Homo sapiens	Burkina Faso
7869191/BF/2016	KY627762	10675	11/05/2016	Homo sapiens	Burkina Faso
904-DHF-10/31/2001	DQ645540	10671	10/31/2001	-N/A-	Taiwan
915-DF-11/03/2001	DQ645541	10616	11/03/2001	-N/A-	Taiwan
950-DF-11/12/2001	DQ645542	10616	11/12/2001	-N/A-	Taiwan
98900663-DHF-DV-2	AB189122	10723	-N/A-	Homo sapiens	Indonesia
98900665-DF-DV-2	AB189123	10723	-N/A-	Homo sapiens	Indonesia
98900666-DSS-DV-2	AB189124	10723	-N/A-	Homo sapiens	Indonesia
BA05i	AY858035	10723	-N/A-	Homo sapiens	Indonesia
D2/Hu/NIID01/2016	LC121816	10685	01/2016	Homo sapiens	-N/A-
D2/Hu/OPD030NIID/2005	LC111438	10678	-N/A-	Homo sapiens	East Timor
D2/Pakistan/2011-23/2011	KF041232	10723	2011	Homo sapiens	Pakistan

D2/Pakistan/2011-3/2011	KF041233	10723	2011	Homo sapiens	Pakistan
D2/Pakistan/2011-4/2011	KF041234	10723	2011	Homo sapiens	Pakistan
D2/Pakistan/209/2009	KF041235	10723	2009	Homo sapiens	Pakistan
D2/Pakistan/51/2008	KF041236	10723	2008	Homo sapiens	Pakistan
D2/Pakistan/78/2009	KF041237	10723	2009	Homo sapiens	Pakistan
D2/Pk/A1/2011	KM217157	10656	08/2011	Homo sapiens	Pakistan
D2/Pk/A2/2011	KM217156	10656	08/2011	Homo sapiens	Pakistan
D2/Pk/Swat-01	KM217158	10656	08/2013	Homo sapiens	Pakistan
D2/SG/05K3295DK1/2005	EU081177	10723	2005	-N/A-	Singapore
D2/SG/05K3330DK1/2005	EU081178	10723	2005	-N/A-	Singapore
D2/SG/05K4137DK1/2005	EU081179	10723	2005	-N/A-	Singapore
D2/SG/05K4155DK1/2005	EU081180	10723	2005	-N/A-	Singapore
D2/SG/CT10/2012	KX380809	10722	2012	Homo sapiens	Singapore
D2/SG/CT12/2012	KX380810	10720	2012	Homo sapiens	Singapore
D2/SG/CT13/2012	KX380811	10723	2012	Homo sapiens	Singapore
D2/SG/CT15/2012	KX380812	10722	2012	Homo sapiens	Singapore
D2/SG/CT18/2012	KX380813	10723	2012	Homo sapiens	Singapore
D2/SG/CT19/2012	KX380814	10677	2012	Homo sapiens	Singapore
D2/SG/CT2/2012	KX380807	10722	2012	Homo sapiens	Singapore
D2/SG/CT20/2012	KX380815	10722	2012	Homo sapiens	Singapore
D2/SG/CT21/2012	KX380816	10719	2012	Homo sapiens	Singapore
D2/SG/CT22/2012	KX380817	10723	2012	Homo sapiens	Singapore
D2/SG/CT23/2012	KX380818	10723	2012	Homo sapiens	Singapore
D2/SG/CT24/2012	KX380819	10722	2012	Homo sapiens	Singapore
D2/SG/CT26/2012	KX380820	10722	2012	Homo sapiens	Singapore
D2/SG/CT27/2012	KX380821	10721	2012	Homo sapiens	Singapore
D2/SG/CT28/2012	KX380822	10722	2012	Homo sapiens	Singapore
D2/SG/CT29/2012	KX380823	10705	2012	Homo sapiens	Singapore
D2/SG/CT30/2012	KX380824	10723	2012	Homo sapiens	Singapore
D2/SG/CT31/2013	KX380825	10722	2013	Homo sapiens	Singapore
D2/SG/CT32/2013	KX380826	10720	2013	Homo sapiens	Singapore
D2/SG/CT34/2013	KX380827	10721	2013	Homo sapiens	Singapore
D2/SG/CT35/2013	KX380828	10723	2013	Homo sapiens	Singapore
D2/SG/CT36/2013	KX380829	10722	2013	Homo sapiens	Singapore
D2/SG/CT38/2013	KX380830	10720	2013	Homo sapiens	Singapore
D2/SG/CT39/2013	KX380831	10718	2013	Homo sapiens	Singapore
D2/SG/CT4/2012	KX380808	10723	2012	Homo sapiens	Singapore
D2/SG/CT40/2013	KX380832	10721	2013	Homo sapiens	Singapore
D2/SG/CT41/2013	KX380833	10722	2013	Homo sapiens	Singapore
D2/SG/CT42/2013	KX380834	10723	2013	Homo sapiens	Singapore

D2/SG/CT44/2013	KX380835	10696	2013	Homo sapiens	Singapore
D2/SG/CT46/2013	KX380836	10719	2013	Homo sapiens	Singapore
D2/SG/CT50/2013	KX380837	10720	2013	Homo sapiens	Singapore
D2/SG/CT51/2013	KX380838	10713	2013	Homo sapiens	Singapore
D2/Taiwan/700TN1509a	KU365903	10638	2015	Homo sapiens	Taiwan
D2/Taiwan/704TN1505a	KU365901	10638	2015	Homo sapiens	Taiwan
D2/Taiwan/704TN1506b	KU365902	10638	2015	Homo sapiens	Taiwan
DC331Y11	KM279515	10723	2011	Homo sapiens	Singapore
DC353Y11	KM279517	10723	2011	Homo sapiens	Singapore
DC357Y11	KM279518	10723	2011	Homo sapiens	Singapore
DC367Y11	KM279519	10723	2011	Homo sapiens	Singapore
DC378Y11	KM279520	10723	2011	Homo sapiens	Singapore
DC380Y11	KM279581	10723	2011	Homo sapiens	Singapore
DC389Y11	KM279582	10723	2011	Homo sapiens	Singapore
DC391Y11	KM279521	10723	2011	Homo sapiens	Singapore
DC395Y11	KM279522	10723	2011	Homo sapiens	Singapore
DC403Y11	KM279523	10723	2011	Homo sapiens	Singapore
DC415Y11	KM279524	10723	2011	Homo sapiens	Singapore
DC427Y11	KM279586	10723	2011	Homo sapiens	Singapore
DC429Y11	KM279525	10723	2011	Homo sapiens	Singapore
DC430Y11	KM279526	10723	2011	Homo sapiens	Singapore
DC589Y12	KM279528	10723	2012	Homo sapiens	Singapore
DC596Y12	KM279587	10723	2012	Homo sapiens	Singapore
DC597Y12	KM279530	10723	2012	Homo sapiens	Singapore
DC618Y12	KM279532	10723	2012	Homo sapiens	Singapore
DC619Y12	KM279533	10723	2012	Homo sapiens	Singapore
DC620Y12	KM279534	10723	2012	Homo sapiens	Singapore
DC621Y12	KM279535	10723	2012	Homo sapiens	Singapore
DC629Y12	KM279536	10723	2012	Homo sapiens	Singapore
DC635Y12	KM279537	10723	2012	Homo sapiens	Singapore
DC636Y12	KM279588	10723	2012	Homo sapiens	Singapore
DC639Y12	KM279538	10723	2012	Homo sapiens	Singapore
DC641Y12	KM279539	10723	2012	Homo sapiens	Singapore
DC642Y12	KM279540	10723	2012	Homo sapiens	Singapore
DC643Y12	KM279541	10723	2012	Homo sapiens	Singapore
DC644Y12	KM279542	10723	2012	Homo sapiens	Singapore
DC645Y12	KM279543	10723	2012	Homo sapiens	Singapore
DC648Y12	KM279571	10723	2012	Homo sapiens	Singapore
DC649Y12	KM279544	10723	2012	Homo sapiens	Singapore
DC652Y12	KM279545	10723	2012	Homo sapiens	Singapore

DC654Y12	KM279546	10723	2012	Homo sapiens	Singapore
DC657Y12	KM279548	10723	2012	Homo sapiens	Singapore
DC661Y12	KM279549	10723	2012	Homo sapiens	Singapore
DC663Y12	KM279550	10723	2012	Homo sapiens	Singapore
DC669Y12	KM279551	10723	2012	Homo sapiens	Singapore
DC673Y12	KM279552	10723	2012	Homo sapiens	Singapore
DC677Y12	KM279553	10723	2012	Homo sapiens	Singapore
DC687Y12	KM279554	10723	2012	Homo sapiens	Singapore
DC688Y12	KM279590	10723	2012	Homo sapiens	Singapore
DC694Y12	KM279555	10723	2012	Homo sapiens	Singapore
DC704Y12	KM279556	10723	2012	Homo sapiens	Singapore
DC710Y12	KM279557	10724	2012	Homo sapiens	Singapore
DC716Y12	KM279591	10723	2012	Homo sapiens	Singapore
DC719Y12	KM279597	10723	2012	Homo sapiens	Singapore
DC720Y12	KM279592	10723	2012	Homo sapiens	Singapore
DC730Y12	KM279558	10723	2012	Homo sapiens	Singapore
DC735Y12	KM279559	10723	2012	Homo sapiens	Singapore
DC740Y12	KM279560	10723	2012	Homo sapiens	Singapore
DC756Y12	KM279561	10723	2012	Homo sapiens	Singapore
DC759Y12	KM279593	10723	2012	Homo sapiens	Singapore
DC763Y12	KM279562	10723	2012	Homo sapiens	Singapore
DC766Y12	KM279563	10723	2012	Homo sapiens	Singapore
DC771Y12	KM279594	10723	2012	Homo sapiens	Singapore
DC786Y12	KM279564	10723	2012	Homo sapiens	Singapore
DC790Y12	KM279565	10723	2012	Homo sapiens	Singapore
DC792Y12	KM279595	10723	2012	Homo sapiens	Singapore
DC793Y12	KM279566	10723	2012	Homo sapiens	Singapore
DC795Y12	KM279572	10723	2012	Homo sapiens	Singapore
DC796Y12	KM279573	10723	2012	Homo sapiens	Singapore
DC811Y12	KM279567	10723	2012	Homo sapiens	Singapore
DC812Y12	KM279568	10723	2012	Homo sapiens	Singapore
DC814Y12	KM279569	10723	2012	Homo sapiens	Singapore
DC827Y12	KM279575	10723	2012	Homo sapiens	Singapore
DC848Y12	KM279570	10723	2012	Homo sapiens	Singapore
DENV-2/BF/BID-V3502/1986	GU131843	10657	1986	Aedes aegypti	Burkina Faso
DENV-2/GU/BID-V2950/2001	HM488257	10967	2001	Homo sapiens	Guam
DENV-2/ID/1016DN/1975	GQ398258	10723	1975	Homo sapiens	Indonesia
DENV-2/ID/1017DN/1976	GQ398259	10723	1976	Homo sapiens	Indonesia
DENV-2/ID/1023DN/1975	GQ398263	10723	1975	Homo sapiens	Indonesia
DENV-2/ID/1046DN/1976	GQ398264	10723	1976	Homo sapiens	Indonesia

DENV-2/ID/1070DN/1976	GQ398260	10723	1976	Homo sapiens	Indonesia
DENV-2/ID/1127DN/1976	GQ398262	10723	1976	Homo sapiens	Indonesia
DENV-2/ID/1172DN/1976	GQ398261	10723	1976	Homo sapiens	Indonesia
DENV-2/IN/BID-V2961/2006	FJ898454	10669	2006	Homo sapiens	India
DENV-2/IND/053598/2005	JQ922551	10639	2005	Homo sapiens	India
DENV-2/IND/969201/1996	JQ922549	10648	1996	Homo sapiens	India
DENV-2/LK/BID-V2416/1996	FJ882602	10677	1996	Homo sapiens	Sri Lanka
DENV-2/LK/BID-V2421/2003	GQ252676	10629	2003	Homo sapiens	Sri Lanka
DENV-2/LK/BID-V2422/2004	GQ252677	10628	2004	Homo sapiens	Sri Lanka
DENV-2/PK	KF360005	10629	11/01/2010	Homo sapiens	Pakistan
DENV-2/PK/2011	KJ010185	10629	10/20/2011	Homo sapiens	Pakistan
DENV-2/PK/2013	KJ010186	10629	10/02/2013	Homo sapiens	Pakistan
DENV-2/Pk/Swat-02	KJ701507	10656	08/27/2013	Homo sapiens	Pakistan
DENV-2/SG/07K3588DK1/2007	GQ398267	10723	2007	Homo sapiens	Singapore
DENV-2/SG/07K3598DK2/2007	GQ398266	10723	2007	Homo sapiens	Singapore
DENV-2/SG/07K3608DK1/2008	GQ398265	10723	2008	Homo sapiens	Singapore
DENV-2/SG/D2Y98P-PP1/2009	JF327392	10723	2009	-N/A-	Singapore
DENV-2/VN/BID-V703/2006	EU482640	10676	2006	Homo sapiens	Viet Nam
DENV-2/VN/BID-V735/2006	EU482672	10678	2006	Homo sapiens	Viet Nam
DENV2-1365	KU509272	10262	2009	Homo sapiens	Thailand
DENV2-14706	KU509277	10551	2010	Homo sapiens	Philippines
DENV2-2627	KU509271	10546	2006	Homo sapiens	India
DENV2-3519	KU509274	10540	2010	Homo sapiens	Philippines
DENV2-3849	KU509275	10455	2008	Homo sapiens	Philippines
DENV2-3850	KU509276	10395	2008	Homo sapiens	Philippines
DENV2-671	KU509268	10523	2009	Homo sapiens	Indonesia
DENV2-973	KU509269	10557	2009	Homo sapiens	Philippines
DENV2-979	KU509270	10536	2012	Homo sapiens	-N/A-
DENV2/CN/GZ05/2014	KP012546	10723	2014	Homo sapiens	China
DENV2/CN/GZ1118/2014	KT187558	10723	2014	Homo sapiens	China
DENV2/CN/GZ25/2014	KP723478	10583	2014	Homo sapiens	China
DENV2/CN/GZ32/2010	KP723479	10723	2010	Homo sapiens	China
DENV2/CN/GZDF31241/2014	KT187553	10723	2014	Homo sapiens	China
DENV2/CN/GZDF571/2014	KT187554	10723	2014	Homo sapiens	China
DENV2/CN/GZDF574/2014	KT187555	10723	2014	Homo sapiens	China
DENV2/CN/GZDF595/2014	KT187556	10723	2014	Homo sapiens	China
DENV2/CN/GZDF615/2014	KT187557	10723	2014	Homo sapiens	China
DS04-221205	EU179858	10709	2005	-N/A-	Brunei
DS09-280106	EU179859	10709	2006	-N/A-	Brunei
DS31-291005	EU179857	10709	2005	-N/A-	Brunei

E1379Y12	KM279598	10723	2012	Homo sapiens	Singapore
E1429Y12	KM279599	10723	2012	Homo sapiens	Singapore
E1432Y12	KM279600	10723	2012	Homo sapiens	Singapore
E1433Y12	KM279576	10723	2012	Homo sapiens	Singapore
E1434Y12	KM279577	10723	2012	Homo sapiens	Singapore
E1436Y12	KM279578	10723	2012	Homo sapiens	Singapore
E1439Y12	KM279601	10723	2012	Homo sapiens	Singapore
E1502Y12	KM279579	10723	2012	Homo sapiens	Singapore
FJ-10	AF276619	10723	-N/A-	-N/A-	China
FJ11/99	AF359579	10723	1999	-N/A-	-N/A-
GD01/01	FJ196852	10723	2001	-N/A-	China
GD01/03	FJ196853	10723	2003	-N/A-	China
GD06/93	FJ196854	10723	1993	-N/A-	China
GD09/93	KC964094	10723	1993	Homo sapiens	China
GWL18 INDI-01	DQ448231	10670	2001	Homo sapiens	India
GZ40	JX470186	10723	2010	Homo sapiens	China
GZ8_12/S/Panyu/2014/DEV2	KX655786	10653	2015	Homo sapiens	China
GZ8_13/S/Yuexiu/2014/DEV2	KX225485	10668	2015	Homo sapiens	China
GZ8_15/S/GZ/2014/DEV2	KX655787	10635	2015	Homo sapiens	China
GZ8_19/S/Haizhu/2014/DEV2	KX225486	10631	2015	Homo sapiens	China
GZ8_70/M/Baiyun/2013/DEV2	KX655788	10668	2015	Homo sapiens	China
ID-CN18-14	KU517846	10723	05/07/2014	Homo sapiens	Indonesia
Jeddah-2014	KJ830750	10718	01/13/2014	Homo sapiens	Saudi Arabia
MKS-0068	KC762665	10723	07/06/2007	Homo sapiens	Indonesia
MKS-0071	KC762669	10723	07/17/2007	Homo sapiens	Indonesia
MKS-0082	KC762658	10723	08/14/2007	Homo sapiens	Indonesia
MKS-0084	KC762660	10723	11/21/2007	Homo sapiens	Indonesia
MKS-0091	KC762670	10723	12/11/2007	Homo sapiens	Indonesia
MKS-0099	KC762676	10723	07/17/2007	Homo sapiens	Indonesia
MKS-0297	KC762662	10723	11/21/2007	Homo sapiens	Indonesia
MKS-0412	KC762661	10723	09/24/2007	Homo sapiens	Indonesia
MKS-0417	KC762655	10723	09/27/2007	Homo sapiens	Indonesia
MKS-0502	KC762656	10723	12/11/2007	Homo sapiens	Indonesia
MKS-2018	KC762673	10723	03/11/2008	Homo sapiens	Indonesia
MKS-2024	KC762663	10723	02/20/2008	Homo sapiens	Indonesia
MKS-2032	KC762675	10723	03/08/2008	Homo sapiens	Indonesia
MKS-2108	KC762671	10723	02/19/2008	Homo sapiens	Indonesia
MKS-2145	KC762664	10723	03/28/2008	Homo sapiens	Indonesia
MKS-2167	KC762674	10723	03/11/2008	Homo sapiens	Indonesia
MKS-2198	KC762672	10723	04/09/2008	Homo sapiens	Indonesia

MKS-2204	KC762667	10723	04/12/2008	Homo sapiens	Indonesia
MKS-2210	KC762659	10723	04/15/2008	Homo sapiens	Indonesia
MKS-2234	KC762657	10723	04/23/2008	Homo sapiens	Indonesia
MKS-3007	KC762666	10723	02/15/2008	Homo sapiens	Indonesia
MKS-IF011	KC762677	10723	02/15/2008	Homo sapiens	Indonesia
MKS-IF014	KC762668	10723	03/11/2008	Homo sapiens	Indonesia
MKS-WS73	KC762678	10723	03/04/2010	Homo sapiens	Indonesia
MKS-WS79a	KC762679	10723	03/29/2010	Homo sapiens	Indonesia
MKS-WS80	KC762680	10723	04/05/2010	Homo sapiens	Indonesia
Od2112	JQ955624	10670	2011	Homo sapiens	India
PG-CN10-13	KU517845	10723	04/23/2013	Homo sapiens	Papua New Guinea
PH-CN77-15	KU517847	10723	08/03/2015	Homo sapiens	Philippines
QHD13CAIQ	KF479233	10723	01/04/2013	Homo sapiens	China
QML16	KX372564	10723	2015	Homo sapiens	Australia
RGCB880/2010	KY427084	10723	09/01/2010	Homo sapiens	India
RGCB921/2011	KY427085	10723	01/03/2011	Homo sapiens	India
RR44	JQ955623	10670	2009	Homo sapiens	India
D2/SG/EHI/03157Y13	KR779786	10723	2013	Homo sapiens	Singapore
D2/SG/EHI/0466Y07	KR779782	10723	2007	Homo sapiens	Singapore
D2/SG/EHI/09087Y15	KY921905	10723	03/2015	Homo sapiens	Singapore
D2/SG/EHI/09423Y14	KY921904	10723	03/2014	Homo sapiens	Singapore
D2/SG/EHI/0017Y06	JN851113	10176	2006	Homo sapiens	Singapore
D2/SG/EHI/0099Y07	GU370050	10200	04/2007	Homo sapiens	Singapore
D2/SG/EHI/0194Y08	JN851119	10176	2008	Homo sapiens	Singapore
D2/SG/EHI/0204Y06	JN851129	10176	2006	Homo sapiens	Singapore
D2/SG/EHI/0232Y06	JN851128	10176	2006	Homo sapiens	Singapore
D2/SG/EHI/0270Y05	JN851130	10176	2005	Homo sapiens	Singapore
D2/SG/EHI/0345Y05	JN851125	10176	2005	Homo sapiens	Singapore
D2/SG/EHI/0377Y04	JN851123	10176	2004	Homo sapiens	Singapore
D2/SG/EHI/0391Y08	JN851118	10176	2008	Homo sapiens	Singapore
D2/SG/EHI/0431Y07	JN851117	10176	2007	Homo sapiens	Singapore
D2/SG/EHI/0462Y05	JN851124	10176	2005	Homo sapiens	Singapore
D2/SG/EHI/0522Y07	JN851114	10176	2007	Homo sapiens	Singapore
D2/SG/EHI/0578Y05	JN851126	10176	2005	Homo sapiens	Singapore
D2/SG/EHI/0615Y08	JN851121	10176	2008	Homo sapiens	Singapore
D2/SG/EHI/0642Y07	JN851115	10176	2007	Homo sapiens	Singapore
D2/SG/EHI/0685Y04	JN851127	10176	2004	Homo sapiens	Singapore
D2/SG/EHI/0722Y07	JN851120	10176	2007	Homo sapiens	Singapore
D2/SG/EHI/0762Y05	JN851131	10176	2005	Homo sapiens	Singapore
D2/SG/EHI/0950Y08	JN851122	10176	2008	Homo sapiens	Singapore

D2/SG/EHI/1079Y07	JN851116	10176	2007	Homo sapiens	Singapore
D2/SG/EHI/1158Y08	GU370051	10527	08/2008	Homo sapiens	Singapore
Taiwan-1008DHF	AY776328	10485	-N/A-	-N/A-	Taiwan
TB16i	AY858036	10723	-N/A-	Homo sapiens	Indonesia
TM101	KX452021	10539	01/2014	Homo sapiens	Malaysia
TM103	KX452022	10667	01/2014	Homo sapiens	Malaysia
TM107	KX452024	10816	01/2014	Homo sapiens	Malaysia
TM109	KX452025	10785	01/2014	Homo sapiens	Malaysia
TM113	KX452026	10674	01/2014	Homo sapiens	Malaysia
TM120	KX452027	10720	01/2014	Homo sapiens	Malaysia
TM121	KX452028	10708	01/2014	Homo sapiens	Malaysia
TM123	KX452029	10627	01/2014	Homo sapiens	Malaysia
TM132	KX452030	10826	01/2014	Homo sapiens	Malaysia
TM135	KX452031	10746	01/2014	Homo sapiens	Malaysia
TM136	KX452032	10780	01/2014	Homo sapiens	Malaysia
TM151	KX452033	10627	01/2014	Homo sapiens	Malaysia
TM16	KX452015	10796	01/2014	Homo sapiens	Malaysia
TM175	KX452034	10615	01/2014	Homo sapiens	Malaysia
TM199	KX452035	10686	01/2014	Homo sapiens	Malaysia
TM203	KX452036	10532	01/2014	Homo sapiens	Malaysia
TM209	KX452037	10615	01/2014	Homo sapiens	Malaysia
TM210	KX452038	10497	01/2014	Homo sapiens	Malaysia
TM220	KX452039	10554	01/2014	Homo sapiens	Malaysia
TM223	KX452040	10665	01/2014	Homo sapiens	Malaysia
TM243	KX452041	10676	01/2014	Homo sapiens	Malaysia
TM244	KX452042	10757	01/2014	Homo sapiens	Malaysia
TM246	KX452043	10741	01/2014	Homo sapiens	Malaysia
TM266	KX452044	10635	01/2014	Homo sapiens	Malaysia
TM280	KX452045	10661	01/2014	Homo sapiens	Malaysia
TM289	KX452046	10600	01/2014	Homo sapiens	Malaysia
TM35	KX452016	10575	01/2014	Homo sapiens	Malaysia
TM38	KX452017	10940	01/2014	Homo sapiens	Malaysia
TM39	KX452018	10807	01/2014	Homo sapiens	Malaysia
TM61	KX452019	10692	01/2014	Homo sapiens	Malaysia
TM74	KX452020	10660	01/2014	Homo sapiens	Malaysia
TSV01	AY037116	10723	-N/A-	-N/A-	Australia
XHZF10615	KY937188	10741	2015	Homo sapiens	China
XLLM10666	KY937186	10742	2015	Homo sapiens	China
XLXY10594	KY937190	10741	2015	Homo sapiens	China
XSMY10589	KY937187	10742	2015	Homo sapiens	China

XZW10694	KY937189	10734	2015	Homo sapiens	China
XZXM10610	KY937185	10742	2015	Homo sapiens	China
ZH1340	EU359009	10723	-N/A-	Homo sapiens	China
ZH413-2	KC131142	10673	09/2012	Homo sapiens	China
ZS01/01	EF051521	10723	2001	-N/A-	China
